# Stereotactic body radiotherapy for localized prostate cancer – 5-year efficacy results

**DOI:** 10.1186/s13014-020-01608-1

**Published:** 2020-07-14

**Authors:** Kristiina Vuolukka, Päivi Auvinen, Erno Tiainen, Jan-Erik Palmgren, Janne Heikkilä, Jan Seppälä, Sirpa Aaltomaa, Vesa Kataja

**Affiliations:** 1 Cancer Center, Kuopio University Hospital, PO Box 100, FI-70029 Kuopio, Finland; 2 University of Eastern Finland, Kuopio, Finland; 3 Department of Urology, Kuopio University Hospital, PO Box 100, FI-70029 Kuopio, Finland; 4 Central Finland Health Care District, Adm Bldg 6/2, FI-40620 Jyväskylä, Finland

**Keywords:** Prostate cancer, Radiation therapy, Stereotactic body radiotherapy

## Abstract

**Background:**

The use of stereotactic body radiotherapy (SBRT) as the primary treatment modality in clinically localized prostate cancer (PCa) is emerging. The aim of the study was to analyze the long-term results of PCa patients treated with SBRT.

**Methods:**

This non-selected, real-life patient cohort included 213 patients with localized PCa treated with a robotic SBRT device during 2012–2015.

**Results:**

The median follow-up was 64 months (range, 10–85 months), and all risk-groups were represented as 47 (22.1%), 56 (26.3%) and 110 (51.6%) patients were classified into D’Amico risk stratification of low, intermediate and high-risk groups, respectively. Androgen deprivation therapy (ADT) was administered to 64.3% of the patients. At cut-off, the biochemical relapse-free survival (bRFS) was 100, 87.5 and 80.0% for patients at low, intermediate and high-risk (*p* = 0.004), and 92.5, 84.2 and 66.7% for patients with Gleason score ≤ 6, 7 and ≥ 8, respectively (*p* = 0.001). The actuarial 5-year overall survival (OS) rates were 97.9, 96.4 and 88.6% in the low, intermediate and high-risk groups, respectively, and at the cut-off, the disease-specific survival (DSS) rate of the whole cohort was high (99.1%), as only two high-risk patients died due to PCa.

**Conclusion:**

Our present results of SBRT delivered with CyberKnife produced excellent long-term bRFS, OS and DSS outcomes among patients with localized PCa. We conclude that SBRT provides an efficient and convenient treatment option for patients with localized PCa, irrespective of the risk-group.

## Background

The use of ultra-hypofractionated (≥ 5 Gy / fraction) external beam radiotherapy (EBRT) is emerging as the primary treatment modality in clinically localized prostate cancer (PCa). It can be delivered as stereotactic body radiotherapy (SBRT) by using an image guided robotic or a gantry-based device. The radiobiology of PCa with its low α/β-ratio and the slow cell proliferation are considered to make PCa sensitive to a high dose per fraction [1–3], and thus hypofractionation advantageous, especially in terms of radiobiological efficacy. Various fractionation schemes have been reported in SBRT literature, but the optimal total dose and dose per fraction are still unknown [4–6].

The majority of the PCa patients included in published SBRT studies have been from low and intermediate-risk groups, with a very good prognosis irrespective of the primary treatment modality [6, 7] and rather few of the patients have been at high-risk [8]. In addition, in most of the studies, the follow-up times have been variable; there are only a few with long follow-up times [9–11]. Because of the increasing use of SBRT, there is an urgent need to determine long-term results in terms of both efficacy and toxicity.

In the US, the published efficacy and toxicity data have been interpreted to be sufficient for listing SBRT in the AUA-ASCO-ASTRO guidelines as an alternative primary treatment option for low-risk patients and an option in research protocols for intermediate-risk PCa patients [12]. With respect to high-risk patients, these guidelines are more cautious, because of the very limited data on the efficacy of SBRT in this group. In Europe, the status of SBRT in the treatment of PCa is controversial. In the PCa treatment guideline issued by the European Association of Urology (EAU), the approach to SBRT is more conservative as its use is restricted only to clinical trials [13] while the European Society for Radiotherapy & Oncology (ESTRO) endorses the AUA-ASCO-ASTRO guideline [14].

The first CyberKnife® device in Scandinavia was implemented in Kuopio University Hospital (KUH) in April 2012 and since then, SBRT has been one of the treatment options for all PCa patients with localized disease, irrespective of their risk-group, who have been referred to RT for their radical primary treatment. The main exclusion criteria for radical intent SBRT have been the cT4 disease, pelvic nodal disease, a previous pelvic RT and previously operated rectal cancer.

The purpose of this retrospective study was to analyze the long-term efficacy results of the new treatment modality among patients with localized PCa. This study focuses especially on the biochemical relapse-free survival (bRFS) and overall survival (OS) according to iPSA, Gleason score and D’Amico risk-stratification.

## Methods

The patient cohort consists of 218 men with biopsy proven and clinically localized PCa who were treated with the robotic SBRT device (CyberKnife® Accuray Inc., Sunnyvale, CA) as their primary curative treatment during April 2012 to March 2015. The diagnosis performed by urologists included PSA testing, digital rectal exam and prostate biopsies and the patients were classified according to D’Amico risk-stratification [15]. The patients with D’Amico high-risk disease were imaged with whole-body computer tomography (CT) and bone scan. At the cut-off in June 2019, five patients had been lost to follow-up, and for the purpose of these analyses, they were excluded. This study has been approved by the Ethics Committee of the Northern-Savo Health Care District (895/13.02.00/2019). The primary endpoint of this study was the long-term efficacy of SBRT in terms of bRFS, disease-specific survival (DSS) and OS among patients with localized low, intermediate and high-risk PCa.

The treatment planning and delivery procedures have previously been described in detail [16]. Briefly, for the purposes of image-guidance, four gold fiducials were placed into the prostate under ultrasound guidance. A non-contrast pelvic CT with 1 mm slice thickness and pelvic magnetic resonance images (MRI) were obtained 7–10 days after the fiducial placement. The MRIs were fused with the CT images to precisely delineate the target volume and the organs at risk (OAR). The clinical target volume (CTV) included the prostate and the proximal seminal vesicles. For high-risk patients with seminal vesicle infiltration (cT3b) in planning MRI scan, the tumorous area of the vesicle was also included into the CTV. To achieve planning target volume (PTV) for patients at low and intermediate-risk, an isotropic expansion of 3–5 mm was added to CTV. The size of the margin was at the discretion of the radiation oncologist according to the accuracies and possible inaccuracies in the target delineation. Posteriorly, the expansion was always only 3 mm to protect the anterior rectal wall. To achieve PTV for high-risk patients, an isotropic expansion of 5 mm was added to CTV, except for 3 mm in the posterior direction. The OARs included rectum, urethra, bladder, bladder wall, femoral heads, penile bulb and testes and the delineation was performed to the planning images according to KUH protocol.

Initially, all patients were treated to the total dose of 36.25 Gy in 5 fractions of 7.25 Gy on every other day delivered with CyberKnife®. In 2013 Katz et al. published a study with a mFU of 60 months showing no additional benefit of the higher total dose of 36.25 Gy as compared to 35 Gy among the low-risk and favorable profile (Gleason 3 + 4 = 7) intermediate-risk patients [17]. Hence, the treatment protocol in KUH was changed and the higher total dose was applied only to patients at intermediate-risk with an unfavorable profile (Gleason 4 + 3 = 7) and patients at high-risk. The prescribed dose was normalized on average to the 85% (range, 76–90%) isodose line. The dose objectives and constraints used for treatment planning are presented in Additional file 1. During a typical 30–45 min treatment session, the fiducials within the prostate were tracked at 15–60 s intervals by using orthogonal X-ray imaging with the robotic treatment device automatically making the necessary adjustments [16]. Administration of androgen deprivation therapy (ADT) was at the urologist’s discretion and based on risk category and prostate size.

During the follow-up time, a typical benign bounce was determined as a prostate-specific antigen (PSA) rise of 0.2 ng/ml or above with a subsequent, spontaneous decline back to the previous nadir or lower. A biochemical relapse (bR) was defined according to the Phoenix definition, i.e. PSA nadir + 2 ng/ml [18] and the bRFS was defined as the time from SBRT to the bR. The DSS was defined as the time from SBRT to PCa death and the OS as the time from SBRT to death due to any cause. In the statistical analyses, the patients were classified into risk groups according to D’Amico risk stratification [15] and the intermediate-risk patients were subdivided into favorable and unfavorable groups according to the Gleason score 3 + 4 = 7 and 4 + 3 = 7, respectively.

All statistical analyses were performed using the SPSS version 22.0 (SPSS Inc., Chicago, IL). The correlations for categorical factors were calculated by Chi-square test, the univariate analyses were conducted with the Kaplan-Meier method, and the significance of the differences between groups was assessed by the log-rank test. Multivariate analyses including the risk-group, PSA at diagnosis (iPSA), Gleason score, the use of ADT and the total dose were performed by Cox-Regression analysis and a *p* value < 0.05 was regarded as statistically significant.

## Results

With a median follow-up (mFU) of 64 months (range, 10–85 months), this study included 213 patients of whom 47 (22.1%), 56 (26.3%) and 110 (51.6%) were classified into low, intermediate and high-risk groups, respectively. The median age of the patients was 70 years (range, 47–86) and the median iPSA was 9.94 ng/ml (range, 1.3–300.0 ng/ml). ADT with varying durations was administered to 137 patients (64.3%). Detailed patient and treatment demographics are presented in Table 1.

**Table 1:** Patient and treatment demographics (*n* = 213)

Patients, *n* = 213	Risk group^a^		
	Low *n* = 47	Intermediate *n* = 56	High *n* = 110
**Age** y			
median	68	69	71
range	52–81	47–86	54–86
**cT-stage** n (%)			
T1c-2a	47 (100)	46 (82.1)	22 (20.0)
T2b	–	9 (16.1)	2 (1.8)
T2c-4	–	–	86 (78.2)
NA	–	1 (1.8)	–
**iPSA ng/ml** n (%)			
< 10	47 (100)	22 (39.3)	39 (35.4)
10–20	–	34 (60.7)	41 (37.3)
> 20	–	-	30 (27.3)
median	7.5	11.1	11.8
range	1.9–10.0	1.3–19.0	2.52–300.0
**Gleason score** n (%)			
≤ 6	47 (100)	27 (48.2)	33 (30.0)
7	–	29 (51.8)	50 (45.5)
≥ 8	–	–	27 (24.5)
**ADT** n (%)			
yes	13 (27.7)	27 (48.2)	97 (88.2)
**ADT duration** n (%)			
7 mo or less	12 (25.5)	17 (30.4)	28 (25.5)
12–18 mo	–	2 (3.6)	13 (11.8)
24 mo or longer	–	7 (12.5)	46 (41.8)
NA	1 (2.1)	1 (1.8)	10 (9.1)
**Irradiation dose** n (%)			
35 Gy	3 (6.4)	10 (17.9)	22 (20.0)
36.25 Gy	44 (93.6)	46 (82.1)	88 (80.0)

^a^D’Amico risk stratification

*Abbreviations*: *iPSA* PSA at the time of diagnosis, *ADT* androgen deprivation therapy, *y* year, *mo* month, *NA* not available

**Fig. 1:**
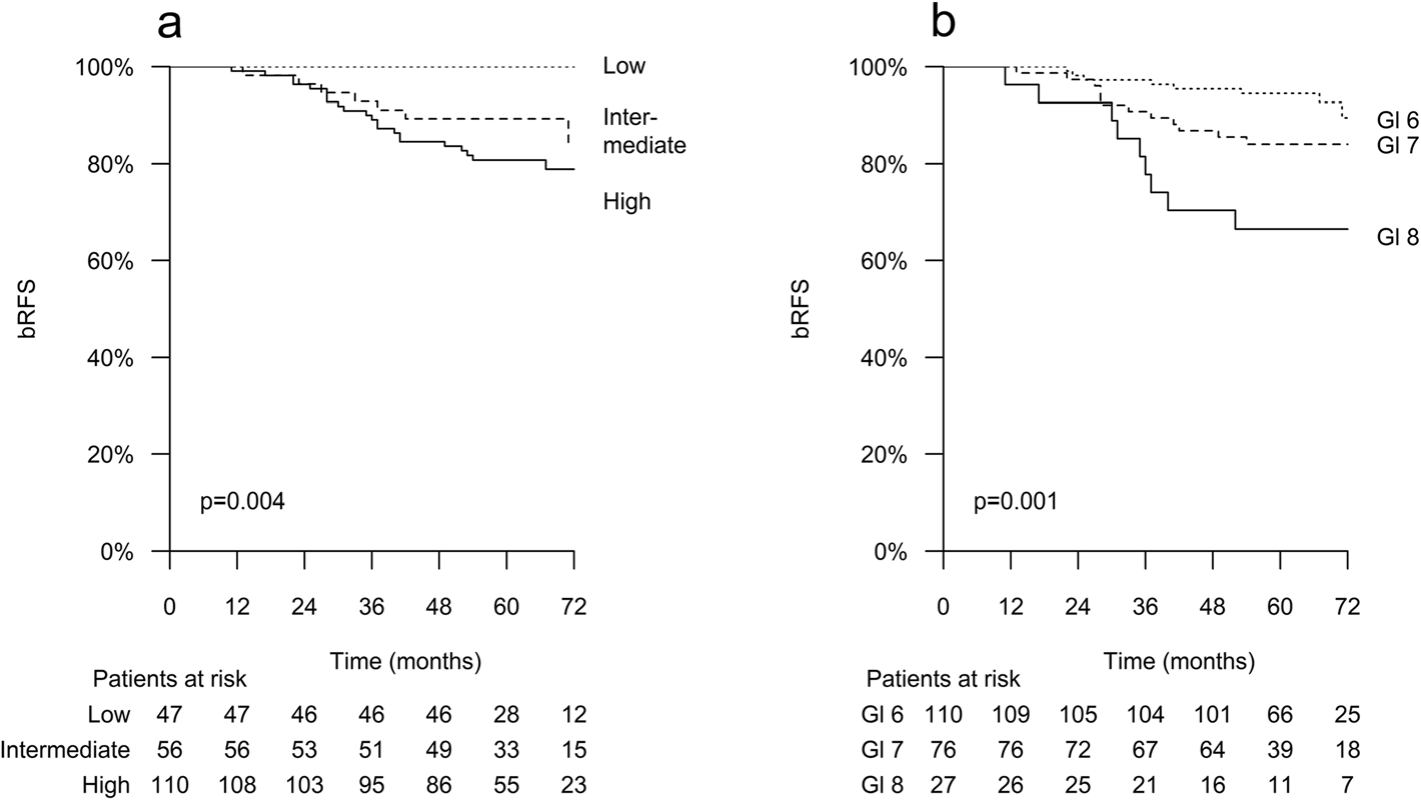
The biochemical relapse-free survival (bRFS) rates at the median follow-up of 64 months according to the risk-group (**a**) and to the Gleason score (**b**)

Biochemical relapse was observed in 29 (13.6%) patients of whom 7 (24.1%) and 22 (75.9%) were classified into intermediate and high-risk groups, respectively, and none in the low-risk group. At mFU of 64 months, the bRFS was 100, 87.5 and 80.0% for patients at low, intermediate and high-risk (*p* = 0.004), and 92.5, 84.2 and 66.7% for Gleason score ≤ 6, 7 and ≥ 8, respectively (*p* = 0.001), as depicted in Fig. 1. The ADT administration correlated significantly with the risk of bR, as the bRFS was 96.1 and 81.0% among the patients treated with ADT compared to those without, respectively (*p* = 0.003). When subdividing the patients into two groups according to the iPSA i.e. less than 10 ng/ml vs 10 ng/ml or higher, the bRFS were 90.7 and 81.7%, respectively, and showing a statistically significant benefit of a low iPSA (*p* = 0.046). The subdivisions into groups of favorable vs unfavorable profile intermediate-risk disease or to the total irradiation dose of 35 Gy vs 36.25 Gy did not reveal any differences between the groups.

The Cox multivariate regression analysis regarding the risk factors for bR was performed including the total RT dose, use of ADT, Gleason score (≤ 7, ≥ 8) and iPSA (≤ 10 ng/ml, ≥ 10 ng/ml). The Gleason score and the use of ADT were found to be significant factors predicting biochemical relapse, see Table 2.

**Table 2:** Multivariate Cox-regression analysis regarding the pretreatment and treatment characteristics and biochemical relapse

Variable	HR (95% CI)	*p* value
Gleason score (≤ 7, ≥ 8)	2.35 (1.03–5.36)	0.043
iPSA (< 10 ng/ml, ≥ 10 ng/ml)	1.36 (0.61–3.01)	0.454
Dose (35 vs. 36.25 Gy)	1.10 (0.41–2.98)	0.846
ADT (no vs. yes)	3.72 (1.06–13.14)	0.041

*Abbreviations*: *Gy* Gray, *iPSA* initial PSA at diagnosis, *ADT* androgen deprivation therapy, *HR* Hazard ratio, *CI* confidence interval

A PSA bounce was observed in 56 (26.3%) patients with a median absolute rise of 0.38 ng/ml (range, 0.2–2.59 ng/ml) with the median time to the first PSA bounce being 17.5 months (range, 6–53 months). Two or more bounces were observed in 9 (4.2%) patients. The development of a PSA bounce correlated with the risk of bR, as the bRFS was 94.6 and 83.4% among the patients experiencing a bounce compared to those without, respectively (*p* = 0.029). Correspondingly, more bounces were seen among patients treated with SBRT only compared to patients treated with SBRT in combination with ADT, 39.5 and 19.0%, respectively (*p* = 0.001). The size (< 50 cm3 vs ≥ 50 cm3) of the prostate did not correlate with the incidence of a bounce and neither did the delivered total RT dose. Among the non-relapsing patients (*n* = 184, 86.4%), the median iPSA of 9.72 ng/ml (range, 1.30–300.00 ng/ml) declined to a median PSA of 0.24 ng/ml (range, 0–3.90 ng/ml), 0.22 ng/ml (range, 0–1.41 ng/ml) and 0.17 ng/ml (range, 0–2.10 ng/ml) at 12, 36 and 60 months, respectively.

At cut-off, the DSS rates were 100, 100 and 98.2% among the patients at low, intermediate and high-risk, respectively. Altogether 18 (8.5%) patients died during the follow-up period; two patients died due to PCa, three due to other malignancies and 13 due to other co-morbidities or trauma. The OS of the whole cohort was 91.5%. The risk group (*p* = 0.203), the Gleason score (*p* = 0.460), favorable or unfavorable profile among the intermediate-risk patients (*p* = 0.902), the iPSA level (*p* = 0.193), the administration of ADT (*p* = 0.242) or the total irradiation dose (*p* = 0.777) did not exert any statistically significant influence on the OS. Interestingly, the development of a bounce correlated significantly with the OS; at the end of follow-up, all the patients (100%) with a bounce were alive as compared to 88.5% of the patients without a bounce (*p* = 0.010). More detailed OS and bRFS results are presented in Table 3.

**Table 3:** Detailed efficacy results according to the risk groups

At mFU of 64 mo	Risk group^**a**^			*p* value*
	Low *n* = 47	Intermediate *n* = 56	High *n* = 110	
**FU** mo				
median	61	64	64	
range	16–83	12–85	10–84	
**bRFS**				
n	47	49	88	
%	100	87.5	80.0	0.004
actuarial 3-y, %	100	92.7	88.8	
actuarial 5-y, %	100	89.1	80.0	
**OS**				
n	45	53	97	
%	95.7	94.6	88.2	0.203
actuarial 5-y, %	97.9	96.4	88.6	

^a^D’Amico risk stratification

*Abbreviations*: *mFU* median follow-up, *mo* month, *bRFS* biochemical relapse-free survival, *y* year, *OS* overall survival

*calculated by log-rank test

## Discussion

This study of robotic SBRT treatment among patients with localized PCa shows excellent results. It seems that SBRT can provide represent an efficient and a convenient treatment option irrespective of the PCa risk-group, as our results compare very well to the results of other primary treatment modalities used in localized PCa [19–25].

In our study, the actuarial 5-year bRFS and OS rates among the high-risk PCa patients were 80.0 and 88.6%, respectively, emphasizing the excellent efficacy of SBRT also among patients with high-risk disease. To the best of our knowledge, this cohort of consecutive high-risk patients is the largest of its kind as the mFU extends over 5 years. There is one published study of a larger pooled cohort (*n* = 125) of high-risk PCa patients treated with 36.25 Gy in five fractions but with a shorter mFU time of 3 years. In that study, the estimated 5-year bRFS of 81% was similar to our results [26]. The longest follow-up is from a single-institution study including 38 high-risk PCa patients; their 8-year biochemical disease-free survival rate was 65.0% [9]. In a review focusing on SBRT in localized high-risk PCa evaluating 13 studies with patient numbers varying from 8 to 125, the reported efficacy results had varying follow-up times and often the results of the high-risk patients were not reported separately [8]. Thus, the results of the present study add valuable knowledge about the efficacy of SBRT in this particular subgroup.

Since the radiobiology of PCa is characterized with a very low α/β-ratio (1.4–1.5 Gy), the total prescribed dose of 35 or 36.25 Gy delivered in less than 2 weeks is comparable to biological 2 Gy equivalent dose of 85–93 Gy. As this theoretical total dose exceeds the total dose of the conventional IMRT treatment at the time (76–78 Gy in KUH), the SBRT protocol was initially adopted also for high-risk PCa patients. The radiobiology of PCa is so unique that it could, at least partly, explain our good efficacy results also among the high-risk patients. This conclusion is supported by the results of a large dose-escalation study of EBRT in which the increase in the total dose correlated statistically significantly with the higher bRFS [20].

In the intermediate-risk group, our actuarial 5-year bRFS of 89.1% is somewhat lower than expected. In the recently published review and meta-analysis conducted by Jackson et al. [6], the median 5-year bRFS of intermediate-risk patients was 92.1%. In addition, Katz et al. have reported 6-year bRFS of 90.7% [17]. Patients with intermediate-risk disease pose a challenge for clinicians, since there is clearly heterogeneity among the aggressiveness of the disease within this group. In attempts to achieve more precise prognostic evaluations, it has been proposed to divide the intermediate-risk group into more specific subgroups according to the tumor burden in biopsies [27] or by applying the new Gleason grading issued by the International Society of Urological Pathology consensus conference in 2014 [28]. In several studies, the subdivision of intermediate-risk group has resulted into bRFS of unfavorable profile patients being closer to the results of high-risk patients [11, 17]. However, in our cohort, the subdivision of intermediate-risk patients into favorable and unfavorable-profile groups according to Gleason score did not reveal any clear division within the group. Unfortunately, in this present study, the data of the tumor burden in the diagnostic biopsies was not known. Perhaps in the future, gene assays tracing prognostic molecular biomarkers could potentially help to identify more aggressive tumors from their indolent counterparts in the intermediate-risk group. After all, in our study with the intermediate-risk patients, the 5-year OS was high (96.4%) and compares well to previous data [11].

The international guidelines are rather unanimous regarding the treatment of SBRT among low-risk patients [12–14, 27]. Our 5-year bRFS, OS and DSS rates of 100, 97.9 and 100%, respectively, underline the excellent prognosis of patients at low risk. Studies reporting long-term efficacy of SBRT in this subgroup include the previously mentioned large pooled analysis conducted by Kishan et al. with 7-year cumulative bR rate of 4.5% and OS rate of 91.4% [11]. The longest reported mFU after SBRT in low-risk PCa is 9 years in a cohort of 230 patients with 10-year biochemical disease-free survival and DSS rates of 93 and 100%, respectively [10]. In this subgroup of patients with a very good prognosis, studies with follow-up times surpassing 10 years are needed to identify the optimal total dosing and fractionation as well as the true efficacy of SBRT.

SBRT for localized PCa is feasible also with modern, image-guided linear accelerators [29, 30]. In previously published data, the acute GU and rectal toxicity have been tolerable with grade 3 adverse events seldom reported and the biochemical control rates have been high [29]. The non-randomized patient cohort published by Nicosia et al. evaluated 149 low and favorable-profile intermediate-risk PCa patients treated to the total doses of 35 and 37.5 Gy delivered in five consecutive fractions of 7 and 7.5 Gy, respectively [31]. With mFU of 33 months, the 3-year biochemical relapse-free survival and OS rates of the whole SBRT cohort were 100 and 96.4%, respectively [31]. Another recent study included 178 patients with low and intermediate-risk PCa with the mFU of 58.9 months [32]. All patients were treated to the total dose of 35 Gy delivered in five fractions on alternate days and ADT was administered to 36 (20.1%) patients. Their 5-year bRFS of 91.6% and OS of 95.1% [32] are in line with our 5-year bRFS rates of 89.1 and 100% and OS rates of 96.4 and 97.9% among low and intermediate-risk patients, respectively.

A recently published randomized controlled trial (HYPO-RT-PC study) compared SBRT and conventionally fractionated EBRT delivered with linear accelerators in localized PCa and the patients represented either the intermediate or high-risk groups [33]. With the 5 years mFU, the SBRT was reported to be non-inferior to conventionally fractionated EBRT, as estimated failure-free survival (FFS) was 84% in both treatment arms [33]. In the HYPO-RT-PC study, all patients were treated without ADT and the SBRT fractionation (42.7 Gy in seven fractions of 7.1 Gy, 3 fractions per week) was different from that used most in robot assisted SBRT studies, likewise in ours. However, their 5-year FFS of 84% was in the same order of magnitude as our 5-year bRFS rates of 89 and 80% among the intermediate and high-risk patients, respectively [33].

The proportion of patients treated with additional ADT was high in our study. Interestingly, this resulted into significantly higher bRFS for the benefit of the combination treatment (*p* = 0.003). In addition, the ADT administration was an independent factor also in the multivariate Cox regression analysis, exerting a strong impact on the risk of bR (*p* = 0.043). Our results differ from previous studies, as in two pooled analyses of SBRT data in localized PCa, the combination of ADT to SBRT has not resulted in a clear benefit [11, 26]. The difference may be due to the high proportion of high-risk patients in our study. When treating intermediate and high-risk patients with conventional EBRT, ADT plays a significant role. The addition of ADT with varying duration has been shown to be beneficial in biochemical control and OS rates in several studies with EBRT to total doses of 65–70 Gy [34] and high-risk patients do benefit from the addition of ADT, even when treated with present-day high-dose EBRT (76–82 Gy) [35]. The role of ADT in combination to SBRT has not been demonstrated before. In the future, it will be a challenging question if it is ethically appropriate to randomize high-risk patients according to ADT in combination to SBRT. Hence, we propose that it is safest to sustain on the ADT recommendations as in combination with EBRT.

The only factor with a statistically significant correlation to OS was the development of a bounce (*p* = 0.010) and it correlated statistically significantly also to the bRFS (*p* = 0.029). In our study with high proportion of patients on hormonal treatment, also the administration of ADT correlated statistically significantly with the bRFS. As ADT itself keeps the PSA-levels very low among castration-sensitive PCa patients, the medication could, at least partly, explain why fewer bounces were seen among patients treated with SBRT in combination to with ADT. Vice versa, patients with low-risk PCa were treated with SBRT only and hence had a higher odds of experiencing a bounce as well as an excellent prognosis according to all efficacy parameters, including OS.

The intrinsic limitation of our study is its retrospective setting. In addition, there was substantial variation in the duration of the ADT administration and unfortunately, the patient reported quality-of-life data has not been systematically collected. The strengths of the study include the real-life nature of the patient cohort, the homogeneity of the RT planning performed by a single treatment-team and the short recruitment time of 3 years. Nevertheless, these results compare well with the efficacy results achieved by other modern treatment modalities available for localized PCa [20, 22, 23, 31, 32, 36].

## Conclusions

In our study, SBRT delivered with CyberKnife® produced excellent 5-year bRFS, DSS and OS rates in all PCa risk groups. These efficacy results were encouraging also among the patients at high-risk and we conclude that SBRT provides an efficient and convenient treatment option for patients with localized PCa in all risk-groups. In the future, the optimal dosing and fractionation of SBRT as well as the role of ADT will need to be determined in randomized studies with sufficient follow-up times.

## Supplementary information

**Additional file 1.** Dose–volume constraints for treatment planning.

## Data Availability

The data that support the findings of this study are available from the patient records of the Kuopio University Hospital, but restrictions apply to the availability of these data, which was used under license for the purposes of the current study only, and so are not publicly available. The data in anonymized form is available from the authors upon reasonable request and with permission of Kuopio University Hospital.

## Abbreviations

ADT: Androgen deprivation therapy
bR: Biochemical relapse
bRFS: Biochemical relapse-free survival
CT: Computer tomography
CTV: Clinical target volume
DSS: Disease-specific survival
EBRT: External beam radiotherapy
ESTRO: European Society for Radiotherapy & Oncology
FFS: Failure-free survival
iPSA: Initial PSA at diagnosis
mFU: Median follow-up
MRI: Magnetic resonance image
OAR: Organ at risk
OS: Overall survival
PCa: Prostate cancer
PSA: Prostate specific antigen
PTV: Planned target volume
SBRT: Stereotactic body radiotherapy
