# Supplementary material for: Stereotactic body radiotherapy for localized prostate cancer – 5-year efficacy results
Source: Radiat Oncol. 2020 Jul 14;15:173. doi: 10.1186/s13014-020-01608-1 (PMC7362647; doi:10.1186/s13014-020-01608-1)
Supplement: Supplementary file 1 — Additional file 1. Dose–volume constraints for treatment planning. [file 13014_2020_1608_MOESM1_ESM.docx]

**Additional file**

Dose-volume constraints for treatment planning.

Prostate V (35 or 36.25 Gy) ≥ 99 %

PTV V (35 or 36.25 Gy) ≥ 95 %

Rectum max dose 38 Gy

V (36.00 Gy) < 1 cc

V (25.00 Gy) < 20 cc

V (36.25 Gy) < 5 %

V (32.63 Gy) < 10 %

V (29.00 Gy) < 20 %

V (18.13 Gy) < 50 %

Bowel V (25.00 Gy) < 1 cc

Bladder max dose 38 Gy

V (37.50 Gy) < 5 cc

V (37.00 Gy) < 10 cc

Bladder wall V (18.30 Gy) < 15 cc

Urethra max dose 40 Gy
